# Managing deliberate self-harm in young people: An evaluation of a training program developed for school welfare staff using a longitudinal research design

**DOI:** 10.1186/1471-244X-8-75

**Published:** 2008-09-15

**Authors:** Jo Robinson, Sara Gook, Hok Pan Yuen, Patrick D McGorry, Alison R Yung

**Affiliations:** 1 Orygen Research Centre, Department of Psychiatry, University of Melbourne, 35 Poplar Road, Parkville, 3052, Australia

## Abstract

**Background:**

Although deliberate self-harm is prevalent among young people, many who engage in deliberate self-harm receive sub-optimal care. Although schools are a well placed setting to support young people who engage in self-harm there are no specific training packages designed to assist school welfare staff to support these young people.

The current study aimed to design, deliver and evaluate a training course specifically for school staff.

**Methods:**

The study employed a longitudinal design. Two hundred and thirteen people participated in the training and evaluation. A questionnaire was administered at baseline, immediately after the training and at 6-month follow-up in order to determine if the training led to improvements in confidence when working with young people who self-harm, perceived skill, knowledge of, and attitudes towards people who self harm.

**Results:**

Prior to the course, the majority of participants demonstrated relatively high levels of confidence, perceived skill and knowledge of self-harm and endorsed relatively positive attitudes towards people who engage in self-harm. Despite this, significant improvements were observed in terms of increased confidence, increased perceptions of skill along with increased knowledge of deliberate self-harm. These improvements were sustained over the follow-up period.

**Conclusion:**

The results demonstrated that the provision of specifically designed training can help school welfare staff to feel better equipped to support young people who are engaging in deliberate self-harm.

## Background

Deliberate self-harm (DSH) as defined below (see Table 1), is prevalent among young people and has recently been identified as one of the primary concerns reported by adolescents in Australia [1]. School surveys report that approximately 5–7% of students aged 15–16 have engaged in self-harm over the previous 12 months, whilst lifetime rates are estimated at 12–13% [2-4]. Deliberate self-harm is one of the strongest known risk factors for suicide [5] and is also associated with an increased risk of further self-harm, accidental death and homicide [3,6].

**Table 1:** The definition of deliberate self-harm employed by the child and adolescent self-harm in Europe group

An act with a non-fatal outcome in which an individual deliberately did one or more of the following:
- Initiated behaviour (for example, self-cutting, jumping from a height), which they intended to cause self-harm;
- Ingested a substance in excess of the prescribed or generally recognised therapeutic dose;
- Ingested a recreational or illicit drug that was an act that the person regarded as self-harm;
- Ingested a non-ingestible substance or object.

Hawton, K., Rodham, K., Evans, E., & Weatherall, R. (2002).

People engage in deliberate self-harm for many reasons. Although a key risk factor is the presence of mental illness [5] many incidents are precipitated by adverse life events, interpersonal crises or self-harm in a friend or peer [2].

Despite the prevalence of DSH among young people, UK studies report that only around 12% of incidents result in a presentation to an Emergency Department [3], and of these, on average only 41% reach the attention of specialist mental health professionals, even for assessment [7]. Failure to receive adequate assessment following self-harm can result in higher rates of repetition than among those who are properly assessed [8,9].

Evidence-based training programmes on managing suicide risk have been found to be effective for example STORM (Skills Training on Risk Management) training [10]. Further, training in Mental Health First Aid has been shown to improve the participants' ability to recognise mental illness, to increase confidence in providing help to someone with a mental illness and to increase the amount of help provided to others [11]. Yet to our knowledge no programs have specifically focused upon deliberate self-harm or been specifically designed for schools.

The current study set out to fill this gap by applying a first aid strategy for deliberate self-harm to schools. This paper reports on the development and evaluation of a training package designed specifically to assist school welfare staff to manage DSH among their students. School welfare staff in Victoria typically refers to school welfare coordinators (often teachers with an additional counselling role), school nurses and school psychologists.

### The present study

The aim of the present study was to evaluate a specifically designed training package that was delivered to school welfare staff across Melbourne and in the Geelong and Warrnambool regions of Victoria in order to determine whether or not the training could improve the participants' ability to provide support to young people engaging in DSH. It was hypothesised that the delivery of the training would lead to: 1) a better understanding of DSH and mental illness 2) an improved ability to recognise risk and mental illness 3) improved levels of confidence and perceived skill in identifying and managing DSH, mental illness and risk amongst participating staff members, and 4) improved attitudes of participants towards young people engaging in DSH.

## Methods

### Research design

The study adopted a single group pre-test/post-test design in order to evaluate a training package designed to assist school welfare staff in the management of DSH among students.

### Setting and sample

ORYGEN Youth Health (OYH) is a public mental health service for people aged 15–24 living in the western and north-western metropolitan regions of Melbourne, Australia. OYH houses an integrated research unit (ORYGEN Research Centre, ORC), and a Mental Health Consultation Program, which provides both training and consultation to school welfare staff on the recognition and management of mental disorders. Secondary schools in the catchment area approached the Mental Health Consultation Program requesting additional support with the management of DSH. As a result the Mental Health Consultation Program partnered with ORC to develop and evaluate a training package for school staff focusing specifically upon the recognition and management of deliberate self-harm. The training package was piloted during 2005. The pilot involved the delivery of one 2-day course to 49 participants from 33 schools. The demand for the training exceeded capacity. A simple course evaluation indicated that participants found the course to be helpful, in particular the sessions on risk assessment and risk management planning. It was then advertised to both public and independent schools throughout Victoria and was delivered to school welfare staff (N = 213) from schools in Melbourne, Geelong and Warrnambool between May and August 2006.

### Procedure

The training program was a 1 or 2-day package (7 hours per day) and participants opted to attend for either 1 or 2 days.

The training was delivered 8 times between May and August 2006. Participants were largely welfare staff from schools in the regions detailed above who opted to attend. All participants were invited to take part in the evaluation. Participants were assessed at 2 time points during the training: baseline (time 1), i.e. immediately prior to the training, and immediately after the training (time 2). Participants were also invited to consent to a 6-month follow-up (time 3). Each questionnaire took around 15 minutes to complete.

### The training intervention

As noted above the training course was delivered over 2 days and participants opted to attend either day 1 or day 1 and 2. Day 1 included the following sessions:

Session 1: A presentation providing up to date information on the epidemiology of DSH and its relationship to suicide and up to date evidence regarding interventions used in school settings.

Session 2: A small group activity using case vignettes during which participants are given the opportunity to explore their attitudes towards DSH, followed by a group discussion.

Session 3: This session focused upon the recognition and assessment of risk. Here participants worked in small groups and using vignettes were asked to consider the individual's level of risk. They were then given some templates of risk assessment tools and asked to role-play conducting a risk assessment. Participants were then shown a DVD of a risk assessment scenario and a group discussion followed.

Session 4: This session focused upon risk management planning. Again the session began with a presentation. Participants then worked in small groups and were given a management-planning template to complete for their case vignette. A group discussion followed.

Session 5: This session discussed the benefits and challenges of working with families. The session took the form of a group discussion followed by a small group activity.

Day 2 began with a brief review of the previous day and then comprised the following:

Session 1: This is a presentation providing up to date evidence on the different type of individual interventions employed when working with young people who engage in DSH.

Session 2: This session provided some basic information about different types of mental disorder and the signs and symptoms to look out for.

Session 3: This session drew upon some of the therapeutic techniques that have been shown to be useful when working with people who self harm. The session began with a presentation and then participants worked in small groups with a case scenario. The group work brought together each of the sessions delivered so far and participants were asked to identify the level of risk, conduct a risk assessment, devise a management plan and to outline some types of interventions that they might consider trying with the young person in their scenario.

Session 4: This session was an opportunity for participants to discuss the policies and procedures that they had in their schools for managing self-harm and to consider how, if at all, these might be improved. Examples of good practice were shared among participants.

Session 5: This session focused upon working with specialist services and took the form of a question and answer session between course participants and representatives from local services.

A brief training resource in the form of a CD ROM was also provided. This contained a PowerPoint presentation summarising the training that was designed to be used by the participants in professional development sessions in their workplaces. No specific training was provided on its use as it was essentially a summary of the training course, however a detailed resource handbook was given to each participant to take back to their workplace which contained an up to date literature review, a copy of all PowerPoint slides used and accompanying training notes.

### Measures

At each time point participants were asked to complete a specifically designed questionnaire, which included questions on demographics, previous experience of, and contact with people with DSH and/or mental illness. They were then assessed in the following areas: confidence, skills, knowledge, attitude towards self-harm and attitude towards suicide prevention. Specifically they were asked four questions, based on those included in the evaluation of the Mental Health First Aid training program [11]:

1. How confident do you feel in helping someone with a mental health (MH) problem?

2. How confident do you feel in helping someone with deliberate self-harm (SH)?

3. How skilled do you feel in helping someone with a mental health (MH) problem?

4. How skilled do you feel in helping someone with deliberate self-harm (SH)?

The participants were to answer the above questions on a scale of 1 to 5 where a score of 1 indicates not at all, a score of 2 indicates a little bit, a score of 3 indicates moderately, a score of 4 indicates quite a bit and a score of 5 indicates extremely. In order to present the results concisely the above scale was converted so that a score of 1 or 2 was categorized as low (L), a score of 3 was categorized as medium (M) and a score of 4 or 5 was categorized as high (H).

They were also asked to complete a series of standardised assessment scales designed to gather information on their level of knowledge of, and attitudes to DSH. These were: the Knowledge of Deliberate Self-harm Questionnaire [12]; the Attitudes Towards Children who Self-Harm Questionnaire [12] and the Attitudes to Suicide Prevention Scale [13]. These outcome measures employ widely different scales. In order to produce a clear and concise presentation of the results, all of these measures were converted into a uniform scale of low, medium and high. This decision was based on the actual ranges of the scores available in the data, the desire to have a simple scale with only a few levels, the need for the resulting scale to be meaningful and evidence from the literature. More details are given below.

The Knowledge of Deliberate Self-Harm Questionnaire (KDS) assessed participants' knowledge of deliberate self-harm. Examples of questions include "Self-harm is more common in girls than boys" and "People who self-harm have an increased likelihood of committing suicide in the future". The score for this measure was the number of correct answers out of 10 given by each participant, thus the allowable range is 0–10. The Attitudes towards Children who Self-harm Questionnaire (ACS) was used to obtain participants' attitudes towards self-harm. Examples of questions in this measure include "My intervention will have no impact on young people who self-harm" and "These children usually make me feel angry". There are 17 items and we asked participants to rate each item as either 'True' or 'False'. For each item, the positive response was given a score of 1 and the negative response a score of 0. An overall score was computed as the sum of the scores of all the items. This overall score has a range of 0 to 17. The items included in the ACS can be categorized into 4 subscales: Effectiveness; Negativity; Worry and Support. The scores of these subscales were again categorized into low, medium and high. To our knowledge, no details regarding the psychometric properties of the KDS and ACS have been published.

Participants' attitudes towards suicide prevention were assessed using The Attitudes to Suicide Prevention Scale (ASP). This scale was selected because although it relates to suicide prevention deliberate self-harm is one of the key risk factors for suicide [5] hence interventions that focus on the reduction or management of self harm can be seen as falling within the scope of suicide prevention activity. The scale includes 14 items (such as "Working with suicidal patients is rewarding" and "If a person survives a suicide attempt then this was a ploy for attention") and participants were asked to rate each item on a 5-point scale from strongly disagree to strongly agree. Again, an overall score was computed as the sum of the scores of all the items, with reverse scoring applied to appropriate items so that a higher score would represent a more positive attitude. The range of the overall score is 14 to 70. The scale's internal consistency is reported as 0.77 and test-retest reliability has been reported as being high with a correlation coefficient of 0.85 (p < 0.001) [13].

As before, the scores of the above-mentioned instruments were converted into low, medium and high categories as indicated in Table 2.

**Table 2:** Categories for the KDS, the attitudes to children who self-harm Questionnaire and the ASP scale

	KDS	ACS	ASP
Low	< 5	< 12	< 46
Medium	5–7	12–14	46–50

High	8–10	15–17	> 50

### Ethical considerations

The Melbourne Health Mental Health Research and Ethics Committee were approached and they informed us that formal ethical approval was not required for this study. All course participants were asked to complete the questionnaires immediately before and after the course as part of the standard evaluation. However written consent was obtained in order that we could contact people again 6 months after the course to obtain longer-term follow-up data.

### Data analysis

Statistical software S-PLUS 6.2 and SPSS Version 12 were used to carry out the analysis. The confidence, skills, knowledge and attitude measures were appropriately divided into low, medium and high levels. At baseline, the percentages of participants falling into each level within each measure were examined. At the post-course time point, we wanted to see if there was a change between baseline and post course, so the McNemar test was applied on the data of these two time points of each categorical measure. At the 6-month time point, we wanted to see if there was stability between post course and 6 months. In other words, we wanted to see if low remained low, medium remained medium and high remained high. This means we wanted to see if there was an association between post course and 6 months. So the Fisher exact test was employed. As an attempt to investigate the effects of some possible covariates on the baseline levels and the change between baseline and post-course, the measures concerned were appropriately dichotomised and logistic regression was employed. A priori a p-value of less than 0.05 was used to determine statistical significance. Further information about the analysis is provided below.

## Results

### Group composition

Of the 213 participants, 199 returned the baseline questionnaire, 185 the post-course questionnaire and 169 (79.3%) returned both. The demographic characteristics of the participants are shown in Table 3. The majority of participants were school welfare staff however there were also some teaching staff and some participants from other settings e.g. government and health organisations. Almost all of the participants indicated that they had worked with someone with either self-harm or a mental health problem (98.5%, N = 194 and 99%, N = 195 respectively), with the mean number of students known to staff to have one of these problems being 10.

**Table 3:** Demographic characteristics

Female (n, %)	171, 85.9
Mean age, SD	42.5, SD 10.6
Received previous training on DSH, suicide risk or mental illness (n, %)	140, 70.7
Mean number of students known to staff to be self-harming or have a mental health problem, SD	10.0, SD 16.8
Place of employment (n, %):	
Government school	98, 47.6
Independent school	48, 23.3
Catholic school	27, 13.1
Education organisation	11, 5.3
Government organisation	11, 5.3
Health organisation	5, 2.4
Other (e.g. employment agency)	6, 2.9

Occupation (n, %):	
Psychologist	55, 28.0
Welfare co-ordinator	40, 20.0
Teacher	29, 15.0
School nurse	23, 11.0
Teaching assistant	2, 1.0
Other (e.g., school counsellor, social worker, chaplain, youth worker)	49, 25.0

### Baseline profile

Overall a minority of participants reported low levels of confidence and perceived skill when working with young people with mental illness and DSH prior to undertaking the training and it appears that participants showed greater confidence and perceived skill when dealing with mental illness than DSH – see Table 4. For the knowledge aspect (KDS), the participants' baseline scores ranged from 2 to 10 (median 7). For the attitude aspects, the baseline ACS total score ranged from 10 to 17 (median 15) and the ASP total score ranged from 43 to 67 (median 53).

**Table 4:** Pre course, post course and follow-up scores: confidence, perceived skill, knowledge and attitudes

		Percentage			Pre vs. Post course: test of change p-value^#^	Post course vs. 6 month: test of stability p-value^†^
				
		Pre course *n = 186–197	Post course *n = 176–182	6 month *n = 74–76		
Confidence MH	L	14.2	2.3	5.3	< 0.001	< 0.001
	M	35.0	22.7	23.7		
	H	50.8	75.0	71.1		
Confidence SH	L	26.9	1.1	3.9	< 0.001	< 0.001
	M	44.7	21.7	25.0		
	H	28.4	77.1	71.1		
Skill MH	L	22.3	4.0	6.7	< 0.001	< 0.001
	M	41.1	26.1	25.3		
	H	36.5	69.9	68.0		
Skill SH	L	34.2	3.4	5.3	< 0.001	0.001
	M	45.4	19.9	26.7		
	H	20.4	76.7	68.0		
KDS	L	2.2	1.7	1.3	< 0.001	0.77
	M	59.7	31.8	28.0		
	H	38.2	66.5	70.7		
ACS	L	3.7	2.2	1.3	0.22	0.004
	M	32.4	38.0	36.0		
	H	63.8	59.8	62.7		
ASP	L	6.4	3.4	5.3	0.015	< 0.001
	M	23.4	15.6	27.6		
	H	70.2	81.0	67.1		
ACS-Effectiveness	L	0.0	0.0	0.0	0.034	0.999
	M	12.7	7.7	5.3		
	H	87.3	92.3	94.7		
ACS-Negativity	L	0.0	0.0	0.0	0.058	0.058
	M	5.4	2.2	2.7		
	H	94.6	97.8	97.3		
ACS-Worry	L	9.7	15.3	9.5	0.12	< 0.001
	M	79.6	75.0	79.7		
	H	10.8	9.7	10.8		
ACS-Support	L	1.0	0.5	0.0	0.71	< 0.001
	M	9.9	9.3	10.7		
	H	89.0	90.1	89.3		

* The sample size for different measures differ slightly at each time point due to missing values.

^# ^Mcnemar test: p < 0.05 means significant change at the 5% level.

^† ^Fisher Exact test: p < 0.05 means significant stability at the 5% level.

L = Low, M = Medium, H = High.

### Change between pre and post test scores (time 1 and time 2)

#### Confidence and skill

There was an overall positive change in terms of confidence and perceived skill in dealing with mental illness and self-harm following the training course (see Table 4). The resulting p-value was less than 0.001 for all analyses indicating that there was a significant change on each of the variables. When looking at the change according to the participants' pre-course levels, we found that the vast majority of those participants who reported low levels of confidence and skill when dealing with mental illness prior to attending the training showed improvement after the course. Similarly, a high percentage of those who were moderately confident showed improvement. As one might expect, those participants who rated themselves as high in confidence and skill remained at this level following the course. The results were similar for confidence and skill when dealing with self-harm. The most improvement occurred among those who reported low and moderate levels of confidence and skill at time 1 – i.e. prior to the course.

#### Knowledge and attitudes

The majority of participants' demonstrated good knowledge of DSH in adolescents and there appeared to be improvement as a result of the training course (see Table 4). Again, when looking at the change according to pre-course levels, we found that all of those who demonstrated a low level of knowledge at the beginning showed improvement, although there were only 3 of them. Seventy eight per cent of those that performed in the moderate range of knowledge improved after the course (73 out of 94). About two-thirds of those with high knowledge showed no change or an improvement following the course (43 out of 60).

Most participants endorsed a positive attitude towards supporting young people who self-harm as shown by the scores on the ACS scale (63.8%, N = 188) and towards their role in preventing suicide among students, as shown by the scores on the ASP scale (70.2%, N = 188). However, the change in attitude between pre and post course is not statistically significant. Of the 14 items in the ASP scale 7 of the items showed a ceiling effect, that is participants scored sufficiently highly at baseline that no improvement could occur (examples of these items include item 1: "I resent being asked to do more about suicide" and item 6: "I feel defensive when people offer me advice about suicide prevention" and item 12: "Suicide prevention measures are a drain on resources"). However participants demonstrated a significant improvement between pre and post course on 2 of the items, these were item 4: "Working with suicidal patients is rewarding" (38.6% to 48.5%, p = 0.006), item 11: "I don't feel comfortable assessing someone for suicide risk" (83.6% to 94.7%, p = 0.001). Item 14 was excluded from the analysis as participants found it hard to answer and as a consequence there were large amounts of missing data (What proportion of suicides do you consider preventable?). This left 4 items where a change would have been possible but was not seen (examples of these include item 2: "Suicide prevention is my responsibility" and item 13: There is no way of knowing who is going to commit suicide").

### Change between post course (time 2) and 6-month follow-up

Follow-up questionnaires were sent to the participants by post 6 months after attendance on the training course in order to determine if the changes shown at the post-course time point would remain at the 6-month time point, i.e. if there was stability between the two time points. Of those participants who consented to be followed up (N = 194) 76 questionnaires were returned, a response rate of 39%. Overall responders and non-responders were similar in terms of their demographic characteristics and on each of the measures assessed at time 1 and time 2.

#### Confidence and skill

The degree of stability between time 2 and time 3 on each of these domains is shown in Table 4. The results of the Fisher exact test indicate that there was significant stability in terms of the confidence and skill measures. It is noteworthy that, at the post-course time point, there are very few people who reported low levels of confidence or skill when dealing with either mental health problems or DSH. Moreover we found that none of these people show any change between time 2 and time 3. There are more people who report moderate levels of confidence and skill and although the numbers are small we found that that whilst changes are reported in each direction, the majority report no change in their confidence and skill levels. Similarly the majority of people who report high levels of confidence and skill show no change over the follow-up period.

#### Knowledge and attitudes

As above, the majority of participants demonstrated moderate or high levels when it came to knowledge of, and attitudes towards DSH and suicide prevention. Table 3 shows the degree of stability on these measures between time 2 and time 3. Here the p value indicates that there is a lack of stability over time, that is the level of knowledge reported at post-test changes between time 2 and time 3 (6 month follow-up). On closer examination it can be seen that whilst 26% (N = 12) of participants who rated at the high level at time 2 were rated at the moderate level at time 3 (i.e. demonstrated a reduction in knowledge), the majority of the variability can be accounted for by the fact that 70% (N = 14) of those participants who were rated at the moderate level at time 2 actually increased in knowledge between time 2 and time 3 (see Table 5).

**Table 5:** Post-course and follow-up scores for the KDS and ACS effectiveness scale

		**KDS 6-month**					**ACS-Eff 6-month**		
					
		**L N (%)**	**M N (%)**	**H N (%)**			**L N (%)**	**M N (%)**	**H N (%)**
**KDS post-course**	**L**	0	0	0	**ACS-Eff post-course**	**L**	0	0	0
	**N = 0**	(0.0%)	(0.0%)	(0.0%)		**N = 0**	(0.0%)	(0.0%)	(0.0%)
	**M**	0	6	14		**M**	0	0	5
	**N = 20**	(0.0%)	(30.0%)	(70.0%)		**N = 5**	(0.0%)	(0.0%)	(100.0%)
	**H**	0	12	34		**H**	0	4	60
	**N = 46**	(0.0%)	(26.1%)	(73.9%)		**N = 64**	(0.0%)	(6.3%)	(93.8%)
			
	**Total**	0	18	48		**Total**	0	4	65
	**N = 66**	(0.0%)	(27.3%)	(72.7%)		**N = 69**	(0.0%)	(5.8%)	(94.2%)

Similarly for the effectiveness subscale of the ACS, the p-value is 0.999, again indicating lack of stability, however as before the majority of this variability is accounted for by the fact that all of the participants (N = 5) who were rated at the moderate level at time 2 were rated at the high level at time 3 – that is they reported increased feelings of effectiveness. This is shown in Table 5.

### Relationship between occupation, course duration, gender and receipt of previous training, and pre and post course assessments

We were interested to examine whether or not any of the following four factors influenced pre and post course assessments: occupation, course duration (1 or 2 days), gender, and previous training. In order to test the effects of the four factors simultaneously, and because a number of the assessments had very few people falling into the low (L) level, two actions were taken to simplify the data. Firstly, occupation was converted into a dichotomized variable: psychologists vs. others, since psychologists made up a quarter of the course participants and it is this group of professionals that we would expect to be up-to-date with issues concerned in the training. Secondly, for each assessment, the M (medium) and L levels were combined into one. In other words, in examining the effects of the four factors, each assessment was treated as a two-level measure: 'L or M' and 'H'. A series of logistic regression analyses were then conducted, this is reported in Table 6.

**Table 6:** Results of logistic regression examining factors affecting pre-course assessments (measured as 'High' vs. 'Low+Medium')

		P-value	OR	95% CI
Confidence MH	Occupation	0.000	3.76	1.85 – 7.65
	Course duration	0.607	1.21	0.59 – 2.49
	Gender	0.230	1.71	0.71 – 4.15
	Previous training	0.001	3.03	1.52 – 6.06
Confidence SH	Occupation	0.012	2.42	1.22 – 4.78
	Course duration	0.958	0.98	0.45 – 2.11
	Gender	0.296	0.62	0.25 – 1.50
	Previous training	0.008	2.88	1.26 – 6.59
Skill MH	Occupation	0.000	5.27	2.64 – 10.53
	Course duration	0.724	1.15	0.53 – 2.49
	Gender	0.434	1.47	0.56 – 3.87
	Previous training	0.002	3.26	1.47 – 7.25
Skill SH	Occupation	0.045	2.17	1.02 – 4.59
	Course duration	0.997	1.00	0.42 – 2.37
	Gender	0.424	0.67	0.25 – 1.77
	Previous training	0.019	3.03	1.11 – 8.28
KDS	Occupation	0.747	0.89	0.45 – 1.78
	Course duration	0.023	0.45	0.22 – 0.90
	Gender	0.574	0.77	0.32 – 1.88
	Previous training	0.950	1.02	0.52 – 2.00
ACS	Occupation	0.312	0.71	0.36 – 1.38
	Course duration	0.602	0.82	0.40 – 1.71
	Gender	0.206	1.75	0.74 – 4.14
	Previous training	0.135	0.59	0.29 – 1.19
ACS-Effectiveness	Occupation	0.747	1.18	0.44 – 3.18
	Course duration	0.404	0.63	0.20 – 1.95
	Gender	0.790	0.84	0.23 – 3.06
	Previous training	0.601	0.77	0.29 – 2.08
ACS-Negativity	Occupation	0.361	0.53	0.14 – 2.01
	Course duration	0.252	2.22	0.59 – 8.35
	Gender	0.842	0.81	0.10 – 6.78
	Previous training	0.935	1.06	0.26 – 4.41
ACS-Worry	Occupation	0.288	0.51	0.14 – 1.84
	Course duration	0.605	0.75	0.26 – 2.16
	Gender	0.826	0.86	0.22 – 3.28
	Previous training	0.023	0.33	0.13 – 0.85
ACS-Support	Occupation	0.315	0.60	0.23 – 1.59
	Course duration	0.092	0.32	0.07 – 1.44
	Gender	0.506	1.52	0.46 – 5.04
	Previous training	0.355	0.59	0.19 – 1.88
ASP	Occupation	0.900	0.95	0.46 – 1.97
	Course duration	0.303	1.48	0.71 – 3.10
	Gender	0.685	0.82	0.32 – 2.12
	Previous training	0.018	2.27	1.15 – 4.46

Occupation: psychologist vs. non-psychologist, Course duration: 2 days vs. 1 day, Gender: Female vs. male, Previous training: Yes vs. No. Reference category of each factor is the second category.

#### Baseline profile

For the perceived confidence and skill assessments, occupation and receipt of previous training appeared to have a significant effect upon both perceived confidence and skill. In particular, psychologists and those who had received previous training in this area were more likely to report greater confidence and skill when dealing with both mental illness and DSH.

With regard to knowledge of deliberate self-harm, course duration was a significant predictor, with those attending a 1-day course demonstrating greater knowledge than those attending both days of the course. Further, those with previous training tended to endorse more positive attitudes towards their role in suicide prevention than those who hadn't had previous training. However previous training was also associated with a greater degree of worry on the 'worry' subscale of the ACS scale; that is those who had received previous training in this area worried more than those who had not.

#### Change between pre and post test scores

Logistic regression was also used to examine factors affecting change pre- and post-course (see Table 7). The findings illustrate that the pre-course level appears to predict the degree of change. That is, those who were rated in the 'low' or 'medium' categories were more likely to show a positive change in all areas. This is unsurprising as those with high pre-test levels could not improve as much as those with low or medium levels due to ceiling effects. Gender also appeared to be a significant predictor for skill in dealing with self-harm, with males having a higher likelihood of showing a positive change.

**Table 7:** Results of logistic regression examining factors affecting change between pre and post-course assessments (measured as 'Positive change' vs. 'No or negative change')

		P-value	OR	95% CI
Confidence MH	Previous level	0.000	0.05	0.02 – 0.14
	Occupation	0.157	0.46	0.16 – 1.36
	Course duration	0.542	1.35	0.51 – 3.58
	Gender	0.210	0.48	0.15 – 1.54
	Previous training	0.604	0.79	0.32 – 1.94
Confidence SH	Previous level	0.000	0.05	0.02 – 0.13
	Occupation	0.839	0.91	0.36 – 2.30
	Course duration	0.203	1.94	0.71 – 5.31
	Gender	0.548	0.68	0.19 – 2.43
	Previous training	0.168	0.49	0.17 – 1.39
Skill MH	Previous level	0.000	0.04	0.01 – 0.11
	Occupation	0.415	0.65	0.24 – 1.80
	Course duration	0.475	1.42	0.55 – 3.69
	Gender	0.057	0.29	0.08 – 1.11
	Previous training	0.443	0.70	0.28 – 1.74
Skill SH	Previous level	0.000	0.02	0.01 – 0.07
	Occupation	0.834	0.89	0.29 – 2.68
	Course duration	0.223	2.11	0.65 – 6.85
	Gender	0.020	0.14	0.02 – 0.85
	Previous training	0.063	0.29	0.07 – 1.18
KDS	Previous level	0.000	0.13	0.06 – 0.28
	Occupation	0.453	0.73	0.32 – 1.66
	Course duration	0.060	2.35	0.96 – 5.76
	Gender	0.417	1.56	0.54 – 4.53
	Previous training	0.275	0.63	0.27 – 1.46
ACS	Previous level	0.000	0.10	0.04 – 0.22
	Occupation	0.312	0.64	0.27 – 1.53
	Course duration	0.739	1.18	0.44 – 3.15
	Gender	0.080	2.82	0.84 – 9.48
	Previous training	0.956	0.97	0.40 – 2.40
ACS-Effectiveness	Previous level	0.000	0.00	X – X
	Occupation	0.719	0.56	0.02 – 12.71
	Course duration	0.355	4.94	X – X
	Gender	0.415	0.00	X – X
	Previous training	0.146	0.00	X – X
ACS-Negativity	Previous level	0.000	0.01	0.00 – 0.07
	Occupation	0.740	1.26	0.33 – 4.90
	Course duration	0.583	1.59	0.28 – 8.93
	Gender	0.184	5.45	X – X
	Previous training	0.723	1.30	0.29 – 5.75
ACS-Worry	Previous level	0.003	0.00	X – X
	Occupation	0.559	0.75	0.29 – 1.96
	Course duration	0.957	0.97	0.35 – 2.74
	Gender	0.824	0.87	0.26 – 2.91
	Previous training	0.359	0.64	0.25 – 1.63
ACS-Support	Previous level	0.000	0.00	X – X
	Occupation	0.860	0.82	0.09 – 7.32
	Course duration	0.264	x	X – X
	Gender	0.245	0.22	0.02 – 3.27
	Previous training	0.252	0.24	0.02 – 3.16
ASP	Previous level	0.000	0.24	0.11 – 0.51
	Occupation	0.457	1.33	0.63 – 2.80
	Course duration	0.712	0.85	0.37 – 1.99
	Gender	0.910	0.95	0.38 – 2.37
	Previous training	0.336	0.69	0.32 – 1.47

Previous level: H vs. L+M, Occupation: psychologist vs. non-psychologist, Course duration: 2 days vs. 1 day, Gender: Female vs. male, Previous training: Yes vs. No. Reference category of each factor is the second category.

X: Unrealistic estimates for odds ratios and confidence intervals due to most subjects showing no change for ACS.

### Training

The follow-up questionnaires also asked a series of questions about people's experience of the training. Participants reported that overall the training had provided them with an understanding of DSH and had given them the opportunity to develop skills in the assessment and management of DSH among students. Seventy-five per cent of participants (N = 57) reported having changed their practice in some way since doing the training and 45% (N = 34) reported wanting additional support in this area. They were also asked if the schools they worked in had a clear policy or set of guidelines in this area to 41% (N = 31) of respondents responded positively.

## Discussion

The purpose of this study was to determine whether or not receipt of a training package specifically designed for school welfare staff improved participants' ability to support young people engaging in DSH. A range of school welfare personnel attended the training. In general, at baseline staff reported relatively high levels of knowledge, confidence and perceived skill when working with these young people, although they reported greater confidence and skill when dealing with mental illness as opposed to deliberate self-harm.

Almost all of the participants had some experience of working with young people who engage in deliberate self-harm (99%) yet less than half report having clear policies or guidelines on the management of DSH in their workplace. This could partially be explained by the fact that the sample was self-selected in that participants elected to attend the course and consented to take part in the evaluation. It would be reasonable to assume that one of the motivations for attending the course was that these professionals have experienced young people with self-harm and felt the need for additional training in this area. In this way the sample may not necessarily be representative of all school welfare staff and may contain inherent biases. The lack of policy or guidelines in this area perhaps warrants further attention.

Overall the training did improve participants' levels of knowledge of, and confidence and perceived skill when working with DSH, in particular among those who reported low levels of knowledge, confidence and skill at the time one assessment and these improvements were largely sustained over the follow-up period.

It was observed that the participants who attended only one day of the course demonstrated greater knowledge at baseline than those attending both days. Perhaps this was because those who did only one day felt more confident and skilled with these issues prior to attending the training and therefore did not feel that they required 2 days worth of training. It was also observed that those who had received previous training in this area worried more about young people who engage in deliberate self-harm than those who had not. This finding was surprising although a similar finding was reported by Crawford and colleagues who found a non-significant trend for participants with more knowledge to be more worried [12]. It may be that those who had received previous training were more aware of what they did not know and realised that they lacked sufficient mental health training to deal with DSH. The others might remain blissfully ignorant. However despite the relatively high levels of confidence and skill reported above we noticed high levels of anxiety among participants throughout the course. Taken together these could indicate that caution should be taken when delivering training in this area, as if it is not thorough enough it might increase, rather than decrease, anxiety in what is already an anxiety provoking area.

## Limitations

There are a number of limitations to the current study, most notably the lack of control group. Whilst there was no reason to assume that the participants' levels of knowledge of self harm or their confidence or perceived skill when working with people who self harm would have changed over this short time period (2 days) in the absence of the training, the repeated measure design adopted by the study means that the effect that has been detected could be a result of either the training intervention or the effect of repeated testing.

Secondly the study sample was not randomly selected. As noted above the sample was self-selected therefore it is not possible to detect whether or not the sample is representative of either schools in the area or school welfare staff. Consequently it is not possible to know whether the improvements seen as a result of the training can be generalised to all school welfare staff. Given the encouraging findings from this study it might be worthwhile to consider further testing the intervention in a larger study that employs a randomised design with a wait-list control group. Thus allowing the above limitations to be addressed.

Thirdly the low consent rate at Time 3 (39%) was disappointing. However, low response rates for postal questionnaires are not uncommon and it was beyond the scope of this study to attempt to increase the response rate using recommended methods such as financial incentives [14].

Finally the study was unable to measure any changes in rates of deliberate self-harm or help-seeking as outcomes of the intervention. In addition the study is unable to detect any changes in practice as a result of the training. However, we have now received funding to continue evaluating the training package for a further year and the new questionnaires include questions about changes in practice; this will be reported in the future.

## Conclusion

The study demonstrated that receipt of a specifically designed training course can improve participants' knowledge, confidence and perceived skill when working with young people with DSH and/or mental illness.

Selective interventions such as educating General Practitioners to better recognise and treat depression, have been shown to be an effective means of reducing suicide risk among the general population [15]. However the effects of other types of gatekeeper training, for example school welfare staff have, to our knowledge, not been subject to rigorous or systematic evaluation. Given that DSH is a key risk factor for suicide and that many (although by no means all) young people present to school welfare staff for help, it is hoped that increasing the capacity of such personnel to recognise and manage mental illness and suicide risk will have a similarly beneficial effect. In addition given that DSH is not only a risk factor for future suicide but is also associated with a range of other negative outcomes, including repeated DSH and forms of premature mortality other than suicide, any intervention that can assist to reduce this behaviour is of merit in its own right.

It has been recommended in suicide prevention that interventions be employed that reflect the whole spectrum of universal, selective and indicated interventions [16]. The current project is just one example of a selective intervention that could be a useful addition when employed in conjunction with other, universal and indicated, suicide prevention initiatives in school settings.

As noted above, we do not know whether the changes evidenced in the current study will translate into improved practice, increased help-seeking or ultimately into a reduction in the rate of deliberate self-harm (and ideally suicide) among students. This was a small, one-year study and it was beyond the scope of the current project to measure these outcomes. Whilst it is not uncommon in suicide research to measure outcomes other than rates of suicidal behaviour itself [17], it is recommended that future research and preventative efforts attempt to measure these key outcomes in order that we can be more certain that interventions such as this are able to meet these broader goals.

## Competing interests

The authors declare that they have no competing interests.

## Authors' contributions

JR conceived of the study, obtained funding, designed the research questionnaires and oversaw the running of the study. JR contributed to data collection and drafted the manuscript. SG assisted with designing the research questionnaires, conducted the majority of data collection, conducted all data entry and contributed to drafting the current manuscript. AY contributed significantly to study design and obtaining funding. AY also contributed significantly to the current manuscript. HPY contributed to the study design and performed all statistical analysis. HPY also contributed significantly to the current manuscript. PM contributed to study design and obtaining funding and to drafting the current manuscript. All authors read and approved the final manuscript.

## Pre-publication history

The pre-publication history for this paper can be accessed here:


